# BDNF/Cyclin D1 Signaling System and Cognitive Performance After Perampanel and Lacosamide Treatment Singly or in Combination in an Experimental Model of Temporal Lobe Epilepsy

**DOI:** 10.3390/cimb46120838

**Published:** 2024-12-11

**Authors:** Michaela Shishmanova-Doseva, Darina Barbutska

**Affiliations:** 1 Department of Pharmacology, Toxicology and Pharmacotherapy, Pharmacy Faculty, Medical University Plovdiv, 4002 Plovdiv, Bulgaria; 2 Research Institute, Medical University Plovdiv, 4002 Plovdiv, Bulgaria; 3 Department of Anatomy, Histology and Embryology, Medical Faculty, Medical University Plovdiv, 4002 Plovdiv, Bulgaria

**Keywords:** perampanel, lacosamide, lithium–pilocarpine, BDNF, cyclin D1, hippocampus, apoptosis

## Abstract

Epilepsy is a common brain function disorder. The present study aims to evaluate the long-term effect of perampanel (PRM) and lacosamide (LCM), administered singly in a high-dose or in a low-dose combination of both, on comorbid anxiety, cognitive impairment, BDNF, and Cyclin D1 hippocampal expression in an experimental model of temporal lobe epilepsy with lithium–pilocarpine. PRM (3 mg/kg, p.o.)/LCM (30 mg/kg, p.o.) or PRM+LCM (0.5 mg/kg + 3 mg/kg, p.o.) treatments were administered three hours after the lithium–pilocarpine-induced status epilepticus and continued for up to ten weeks in adult Wistar rats. Our study demonstrated that perampanel and lacosamide administered singly in high doses improved epilepsy-associated cognitive impairment through ameliorating anxiety and facilitating passive learning and memory, with spatial and recognition memory measured in the elevated plus maze, step-through, Y-maze, and object recognition tests, respectively. In addition, the combination of both drugs in low doses demonstrated similar anxiolytic and cognitive-improving effects compared to the singly administered drugs. Moreover, the three experimental groups enhanced the hippocampal expression of the neurotrophic factor BDNF and mitigated the increased levels of the apoptotic factor Cyclin D1. These beneficial effects could be essential mechanisms through which administered anticonvulsants preserve neuronal survival and homeostasis in the CNS and especially in the hippocampus.

## 1. Introduction

Epilepsy is one of the most common brain function disorders, and most focal epilepsy crises arise in the temporal lobe of the brain [1,2]. Although the pathogenesis of epilepsy is still not fully elucidated, different molecular targets, such as neurotransmitters, ion channels, or synaptic proteins [3], can be influenced by existing anti-seizure medications (ASMs). It is well known that glutamate is the primary excitatory neurotransmitter in the central nervous system (CNS), and hyperactivation of its postsynaptic α-amino-3-hydroxy-5-methylisoxazole-4-propionic acid (AMPA) receptors plays a crucial role in the generation, synchronization, and spread of epileptic activity [4,5]. Perampanel (PRM) is a novel third-generation ASM. It is the first drug that belongs to the class of non-competitive, selective AMPA receptor antagonists [6]. PRM has been approved as an adjunctive treatment for focal epileptic seizures with or without secondary generalization in patients with epilepsy aged ≥ 4 years and for primary generalized tonic–clonic seizures in patients with idiopathic generalized epilepsy aged ≥7 years (EU only) or ≥12 years (USA only). In the USA, the drug can be prescribed as a monotherapy for the treatment of focal epileptic seizures [7].

Although most ASMs act on voltage-gated sodium channels, lacosamide (LCM) is a third-generation drug with a unique mechanism of action on these channels. It selectively enhances their slow inactivation by regulating their long-term availability [8]. Voltage-gated sodium channels are responsible for the generation of action potentials during normal brain function and high-frequency firing, which is a feature of epileptic discharges [9]. LCM has been approved for the treatment of focal seizures with or without secondary generalization as a monotherapy or an adjunctive drug for patients ≥16 years of age [10].

Different comorbidities, including cognitive deficits, depression, anxiety, migraine, and a tendency for falls and fractures, can be observed among epileptic patients [11]. Numerous ASMs have been shown to be clinically effective [3]. Despite the latter fact, more than 30% of patients remain refractory to seizure treatment, which requires the prescribing of two or more ASMs [12]. Combinations of different anticonvulsants can produce additive, synergistic, or antagonistic effects on seizure control, alongside some adverse effects [13]. From a pharmacological perspective, polytherapy includes two or more ASMs with different mechanisms of action that influence various targets for seizure control. Considering the large number of anticonvulsants available, the consequences of different drug combinations have, in general, not been well elucidated.

At this stage, there are not enough studies related to the influence of epileptic seizures on neuronal survival in the hippocampus. There is also a lack of research on the possibility that anticonvulsant drugs affect the expression of neurotrophic factors in the hippocampus, a brain structure extremely critical for memorizing and consolidating information from short- to long-term memory. Brain-derived neurotrophic factor (BDNF) is an endogenous protein that is a key regulator of brain development and neuronal formation. It promotes neurogenesis and synaptic activity, protects neurons from programmed cell death, and maintains cellular homeostasis [14,15,16]. In contrast, BDNF is a mediator of neuron-microglial interactions [17,18]. Many researchers consider BDNF to be the most important signaling molecule between neurons and glia, especially in conditions of injury and cellular stress [19]. According to several studies, microglia express BDNF, and in turn, the derived BDNF affects synaptic plasticity, influencing different neuronal structures and functions [20,21]. The released neurotrophic factor can affect neuronal survival and compensate for neuronal loss [20,21]. Whether and how these interactions are unidirectional and what their subtle mechanisms are is a question that has not been sufficiently investigated.

In recent years, there has been evidence of a cascade of processes occurring in cells as a result of active external factors (cellular stress, damage, and intake of certain drugs), such as the process of apoptosis. The latter is a complex process regulated by multiple pro- and anti-apoptotic genes; long-term damage to the balance between the two groups causes apoptotic changes in the brain cells [22]. The hippocampus is extremely sensitive to all external influences that cause oxidative stress in cells [23]. In this respect, the participation of programmed cell death in the mechanisms of epilepsy, as well as the causes leading to cognitive deficits, are also considered. The Cyclin D1 protein participates as a regulator of some manifestations of the apoptotic process. Moreover, increased expression of this protein has been revealed in hippocampal neurons and proliferating glia after epileptic seizures [24,25]. Therefore, Cyclin D1 has been a subject of research in two lines of investigation: participation in the cell cycle and provoking apoptotic changes in major damage to the cell’s DNA [26]. The data suggests that Cyclin D1 is a convenient marker for detecting probable neuronal damage in the presence of apoptotic cell changes. Cyclin D1 is visualized histochemically in damaged neurons of the hippocampus and in some glial elements [27]. The probable control of neuronal functions and neurogenesis by astrocytes through the expression of Cyclin D1 has also been described [28].

Animal models of epilepsy have proven to be invaluable in the evaluation of ASMs on comorbidities and the possible mechanisms through which drugs affect these comorbidities [9]. The lithium–pilocarpine model is a reliable animal model for investigating epileptogenesis after status epilepticus (SE) and pathogenesis of temporal lobe epilepsy (TLE), which is demonstrated by spontaneous motor seizures (SMS) and comorbid cognitive and psychological disorders during the chronic phase [2]. In this respect, we evaluated the long-term effect of PRM and LCM, administered singly in a high-dose or in a low-dose combination of both on comorbid anxiety, cognitive impairment, BDNF, and Cyclin D1 hippocampal expression in an experimental model of TLE.

## 2. Materials and Methods

### 2.1. Reagents and Drugs

Pilocarpine hydrochloride (Pilo, powder, P6503), lithium chloride (LiCl, powder, L9650), and scopolamine methyl bromide (Scop, powder, S8502) were purchased from Sigma Aldrich, St. Louis, MO, USA. Lacosamide (Vimpat^®^, USB Pharma, Brussels, Belgium), Perampanel (Fycompa^®^, Eisai Ltd., Frankfurt am Main, Germany), and Diazepam (Sopharma, Sofia, Bulgaria) were obtained from the pharmaceutical companies. The following reagents were used in the study: Bouen’s Fluid Sigma, HT10132 (Aldrich Chemie GmbH, Taufkirchen, Germany); Gill’s Hematoxylin Solution, No. 2, sc-24973A (Santa Cruz Biotechnology, Inc., Dallas, TX, USA); ImmunoCruz goat ABC Staining, sc-2023 (Santa Cruz Biotechnology, Inc., Dallas, TX, USA); pro BDNF (5H8), sc-65514; Cyclin D1 (A-12), sc-8396 (Santa Cruz Biotechnology, Inc., Dallas, TX, USA); and VectaMount^®^ Express Mounting Medium, H-5700-60 (Vector Laboratories, Newark, CA, USA).

### 2.2. Animals

Forty-six mature male Wistar rats at 3 months of age weighing between 180 and 200 g were used in this study. They were obtained from the Animal Center of the Medical University, Plovdiv. The rats were housed in plastic cages (4–5 per cage) [29,30,31]. They were maintained at a constant temperature (22 ± 1 °C) and relative humidity (55–60%) under a 12 h/12 h light/dark cycle. The ventilation system was set between 15 and 20 air changes per hour. Food and drinking water were provided ad libitum. This study was performed in strict accordance with the guidelines of European Community Council Directives 86/609/EEC and 0.2010/63/EC. The experiments were approved by the Bulgarian Food Safety Agency No. 206/01.10.2018 and the Ethical Committee on Human and Animal Experimentation of Medical University—Plovdiv No. 1/28.02.2019.

### 2.3. Generation of Chronic Epilepsy Rats

**Figure 1:**
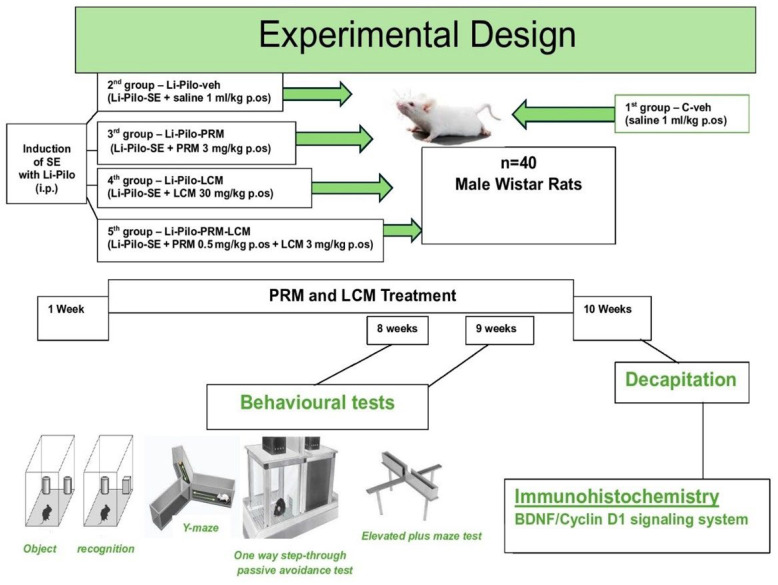
Timeline of the experiment.

The timeline of the experiment is shown in Figure 1. Thirty-eight rats were injected with LiCl 127 mg/kg intraperitoneally (i.p.) 24 h before the Pilo treatment. The rats were given 30 mg/kg i.p. Pilo, 30 min after the injection of scopolamine methyl bromide 1 mg/kg i.p. The latter was administered to reduce the peripheral cholinergic effects resulting in respiratory problems, which are one of the main reasons for the animal’s death in this model. After the last injection, the animals were placed in separate cages, and their behavioral seizure severity was evaluated based on the Racine scale [32]: Stage 1—mouth and facial movements; Stage 2—head nodding; Stage 3—forelimb clonus; Stage 4—seizures characterized by rearing and continued forelimb clonus; Stage 5—seizures characterized by rearing, forelimb clonus, and falling. The criterion for reaching status epilepticus was a state when the animal reached stage 4 or 5 based on the Racine scale, followed by continuous motor activity (>9 motor seizures/h). After two hours of ongoing seizures, 10 mg/kg Diazepam was injected i.p. to relieve convulsions and prevent mortality. The dosage was repeated as needed. Six animals died during the SE. The remaining 32 rats were then randomly allocated to the four experimental groups (8 per group, from the second to the fifth one), and an additional 8 rats, in which no SE was induced, were allocated to the control group as follows:

◦ 1st group—C-veh (control animals treated only with saline 1 mL/kg p.os.);
◦ 2nd group—Li-Pilo-veh (this group was with the model of post-SE-induced TLE triggered by the i.p. administration of LiCl 127 mg/kg and Pilocarpine 30 mg/kg i.p. and was treated with saline 1 mL/kg p.os.);
◦ 3rd group—Li-Pilo-PRM (this group was with the model of post-SE-induced TLE triggered by the i.p. administration of LiCl 127 mg/kg and Pilocarpine 30 mg/kg i.p. and was treated with perampanel 3 mg/kg p.os.);
◦ 4th group—Li-Pilo-LCM (this group was with the model of post-SE-induced TLE triggered by the i.p. administration of LiCl 127 mg/kg and Pilocarpine 30 mg/kg i.p. and was treated with lacosamide 30 mg/kg p.os.);
◦ 5th group—Li-Pilo-PRM-LCM (this group was with the model of post-SE-induced TLE triggered by the i.p. administration of LiCl 127 mg/kg and Pilocarpine 30 mg/kg i.p. and was treated with perampanel 0.5 mg/kg p.os + lacosamide 3 mg/kg p.os).

Three hours after the Li-Pilo injections, the rats were injected subcutaneously (s.c.) with NaCl 0.9% and glucose 5% in equal volumes (up to 3% of body weight) to restore volume loss. This procedure was repeated over the next 5 days to increase the survival chances of the animals. Treatment with veh/PRM or LCM, administered orally by gavage, started three hours after the onset of SE and continued for up to 10 weeks. We used the pharmaceutically prepared solution for infusion of lacosamide (Vimpat^®^ 200 mg/20 mL). Doses of 3 mg/kg (0.03 mL per 100 g) and 30 mg/kg (0.3 mL per 100 g) were administered per os from this solution. Perampanel (Fycompa 2 mg tab.) was dissolved in a three-component solvent (distilled water, DMSO, and propylene glycol) in a concentration of 1 mg/10 mL. Doses of 0.5 mg/kg (0.5 mL per 100 g) and 3 mg/kg (3 mL per 100 g) were administered per os [33]. Eight weeks after the Li-Pilo injection, all rats were subjected to a series of tests and were daily treated with the drugs.

### 2.4. Behavioral Tests

#### 2.4.1. Object Recognition Test

The object recognition test (ORT) consisted of a training session (T1) and a test session (T2) conducted on two consecutive days. The object recognition test was performed in an open Plexiglas box (60 × 60 × 40 cm) with objects made of plastic, as previously described [34,35]. Briefly, during T1, each rat was allowed to explore two identical objects (A1 and A2) for 5 min. In the test session (T2), the rats were exposed to a familiar object (A1) and a novel object (B) for 5 min. The exploration time (s) for each object in the test session was recorded, and the discrimination index (DI) was defined as the difference in exploration time between the novel (TB) and familiar objects (TA) divided by the total time spent exploring the two objects in the discrimination phase: (TB − TA)/(TB + TA) = DI.

#### 2.4.2. Y-Maze

The Y-maze is widely used to assess hippocampal-dependent spatial working memory by measuring the spontaneous alternations. This method utilizes the congenital tendency of rodents to explore novelty [36]. Black Plexiglas consisting of three equally spaced arms interconnected at 120° was used in the experiment. Each arm was designated as A, B, or C. At the beginning of the experiment, each animal was randomly placed at the end of one arm of the maze and allowed to explore the maze freely for a total trial duration of 7 min. Successful entry into an arm was considered when the animal entered the arm on all four paws. The number and sequence of arm entries were recorded manually. Spontaneous alternation occurs when the animal consecutively enters at least three of various combinations of arms; for example, ABC, CBA, CAB, BCA, etc. Spontaneous alternation (SA) % was calculated using the following formula: SA % = (number of alternations)/(total number of entries − 2) × 100.

#### 2.4.3. One-Way Step-Through Passive Avoidance Test with Negative Reinforcement

The step-through apparatus (UgoBasile, Gemonio, Italy) consisted of two equal-sized dark and light compartments separated by a sliding automatic door. The experiment was conducted on two consecutive days: the 1st day was the learning session, which was performed with a schedule of 2 trainings with a 60 min interval between them, and the 2nd day was the retention test. During the learning session, the rats were placed in the light compartment. Immediately after the rat entered the dark chamber with all four feet inside, the door was closed and a short-lasting electrical foot shock (9 s, 0.4 mA, 50 Hz) was delivered. After 20 s, the animals were removed from the dark chamber and returned to their cages. Time (s) spent in the light compartment was recorded. Twenty-four hours after the last learning session, the memory retention test was performed. The experimental protocol was the same as above, except that no electrical shock was delivered to the rats. The test was concluded when the animal entered the dark compartment or remained in the light chamber for 178 s (cut-off time) [37].

#### 2.4.4. Elevated Plus Maze Test

The apparatus consisted of two open arms (50 × 10 cm^2^), two enclosed arms (50 × 10 × 50 cm^3^), and a central platform (10 × 10 cm^2^), elevated 50 cm above the floor. Rats were placed on the central square of the maze facing an open arm and allowed to freely explore the maze for 5 min. The calculated measures were: (1) the number of entries in open arms; (2) the number of entries in enclosed arms; (3) the time (s) spent in the open arms; and (4) the anxiety index (AI) = 1 − [(open arms time/total time) + (number of entries in open arms/total number of entries)/2]. Results close to 1 show anxious behavior [34,35].

After the last test, animals were decapitated under deep anesthesia with 0.2 mg/kg, i.p. of fentanyl and 0.3 mg/kg, i.p, of midazolam. Brains were removed immediately and collected for further investigations. Parts of the brains of experimental animals containing the hippocampus were fixed using Bouen’s fluid for 24 h. After washing with xylol, the brain fragments stayed in liquid paraffin in a thermostat (at 56°) for 24 h, after which they were incorporated into paraffin. The paraffin blocks were cut into an automatic paraffin microtome (Leica 2055, Leica Biosystems, Deer Park, IL, USA). Paraffin histological sections (6 μm thick) were installed on silane-coated slides and were used for immunohistochemical analysis.

### 2.5. Immunohistochemistry

We used fragments of the hippocampus of the experimental animals, processed by protocol, included in paraffin, and prepared for immunohistochemical analysis. The cuts were mounted on silane-coated slides. The slides were dewaxed alternately with xylene and a descending alcohol battery. Then, we suppressed peroxidase with a solution of methanol and hydrogen peroxide for 30 min. The slides were kept in PBS (phosphate-buffered saline) for 15 min and in normal swine serum dissolved in PBS for 30 min. The expression of BDNF and Cyclin D1 was monitored using the ABC method with the ImmunoCruz™ rabbit ABC Staining System kit (Santa Cruz Biotechnology, Inc., USA), with DAB as chromogen and monoclonal primary anti-BDNF and anti-Cyclin D1 antibody (1:100; Santa Cruz Biotechnology, Inc., USA) [38,39]. Sections in which the primary antibody was substituted with PBS were used as negative controls. The duration of incubation was 24 h at 4° C in a humid (70%) chamber. After completion of the reaction, we rinsed with PBS, contrasted the nuclei with hematoxylin, dehydrated in alcohols of upward concentration, and covered with Vecta mount (Vector Laboratories, Newark, CA, USA).

The immunohistochemical manifestations of the investigated signal system are subjected to quantitative analysis of the intensity of reactions using specialized software, the Leica DM 3000 image system with Flexacam C3 (Leica Biosystems, Deer Park, IL, USA).

### 2.6. Quantitative Analysis of the Intensity of Immunohistochemical Reactions

The immunohistochemical analysis was performed on both brain hemispheres of all animals from the groups (n = 40), using specialized, highly sensitive software, the Olympus DP-Soft image system (version 4.1 for Windows), equipped with a Camedia-5050Z digital camera (Olympus, Japan). The intensity values of the reactions were in the range of 0–256, with 0 presenting white and 256 black. The specific staining of the fields was in the form of dark brown to black granules in the positive cells. The analysis was carried out on sections of the hippocampus of Wistar rats from all groups already described (n = 40). Series of simultaneous reactions involving material from all experimental groups were analyzed in order to determine the comparability of the results. The curve of the intensity of immune responses in the positive cells was calculated on different microscopic fields of sections (20 sections per animal, magnification ×400) of all hippocampal hemispheres of at least 100 cells. The mean value of the intensity of antigen expression for each animal in the groups was calculated, and BDNF/Cyclin D1 expression was recorded in relative units (RU).

### 2.7. Statistical Analysis

Experimental results were presented as mean ± SEM. The results were analyzed using parametric tests because of normally distributed data, as assessed by the Kolmogorov–Smirnov test. The different parameters of all behavioral tests—discrimination index, spontaneous alternations, step-through latency time, number of entries in open arms, time spent in open arms, and anxiety index, including BDNF and Cyclin D1 hippocampal expression—were assessed by one-way ANOVA. When the F-ratio was significant, the between-group differences were assessed by Tukey’s post hoc test in case of justification. When the variances were significantly different, depending on the homogeneity of the dispersions (found by using the Levene’s test), the Games–Howell post hoc test was applied. Statistical significance was set at *p* < 0.05. The analysis was conducted by using the IBM SPSS^®^ (version 19.0.) statistical package.

## 3. Results

### 3.1. Object Recognition Test

**Figure 2:**
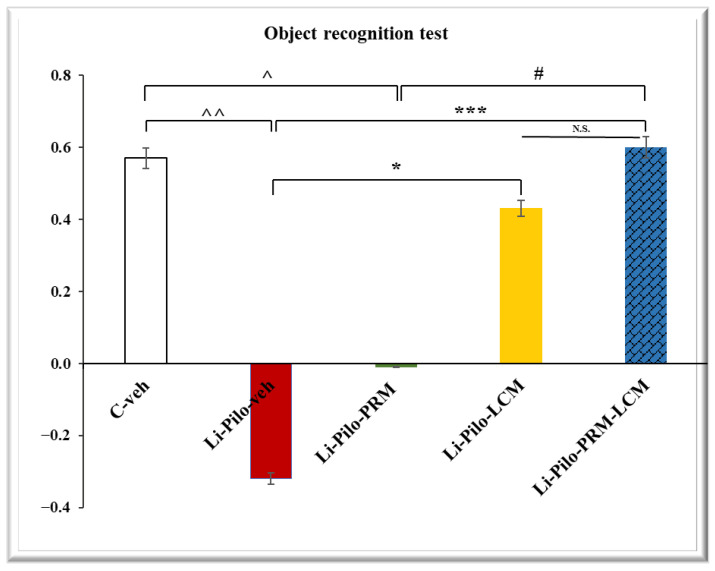
Effect of long-term treatment with perampanel (PRM) and lacosamide (LCM), singly or in combination, on the discrimination index in the object recognition test in animals with a model of temporal lobe epilepsy. ^^^^ *p* < 0.01 Li-Pilo-veh group vs. C-veh group; ^^^ *p* < 0.05 Li-Pilo-PRM vs. C-veh group, * *p* < 0.05 Li-Pilo-LCM vs. Li-Pilo-veh group; *** *p* < 0.001 Li-Pilo-PRM-LCM vs. Li-Pilo-veh group; ^#^ *p* < 0.05 Li-Pilo-PRM-LCM vs. Li-Pilo-PRM group; no significance (N.S.), *p* > 0.05 Li-Pilo-LCM vs. Li-Pilo-PRM-LCM group.

The post hoc test demonstrated that the Li-Pilo-veh group had impaired object recognition memory compared to the C–veh group (−0.31 ± 0.09 vs. 0.57 ± 0.08, *p* < 0.001), while the long-term LCM treatment during the chronic phase of the TLE model managed to increase the discrimination index compared to the group with epilepsy (0.43 ± 0.09 vs. −0.31 ± 0.09, *p* = 0.001) (Figure 2). The Li-Pilo-PRM group singly did not increase the discrimination index, while the group treated with both drugs, PRM and LCM, managed to reduce the negative influence of epilepsy compared to the pilocarpine-treated animals (0.6 ± 0.09 vs. −0.31 ± 0.09, *p* = 0.001) and to the rats treated only with PRM (0.6 ± 0.09 vs. −0.01 ± 0.2, *p* = 0.009). No statistical significance was observed when the C-veh group was compared to the Li-Pilo-LCM and Li-Pilo-PRM-LCM animals as well as between the Li-Pilo-LCM and Li-Pilo-PRM-LCM rats.

### 3.2. Y-Maze Test

**Figure 3:**
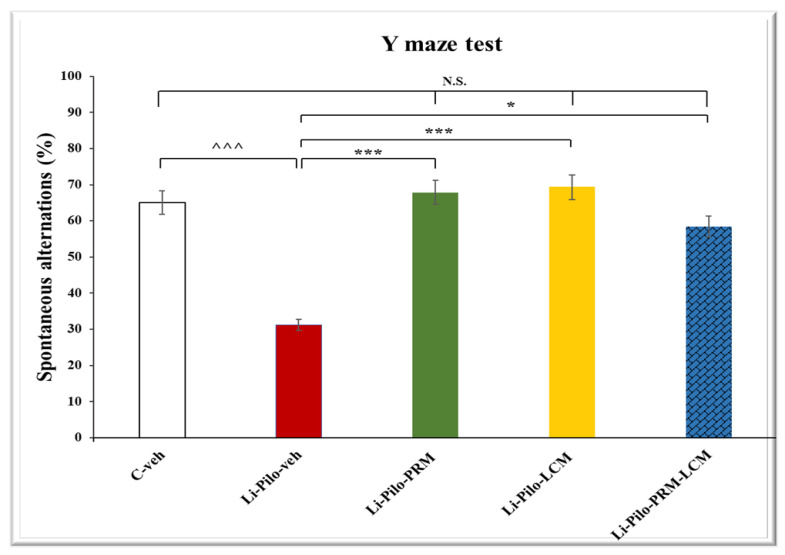
Effect of long-term treatment with perampanel (PRM) and lacosamide (LCM), singly or in combination, on the spontaneous alternations (%) in the Y-maze test in animals with a model of temporal lobe epilepsy. ^^^^^ *p* < 0.001 Li-Pilo-veh vs. C-veh group; *** *p* < 0.001 Li-Pilo-PRM vs. Li-Pilo-veh group; *** *p* < 0.001 Li-Pilo-LCM vs. Li-Pilo-veh group; * *p* < 0.05 Li-Pilo-PRM-LCM vs. Li-Pilo-veh group; no significance (N.S.), *p* > 0.05 C-veh group vs. Li-Pilo-PRM group; *p* > 0.05 C-veh group vs. Li-Pilo-LCM group; *p* > 0.05 C-veh group vs. Li-Pilo-PRM-LCM group; N.S., *p* > 0.05 Li-Pilo-PRM vs. Li-Pilo-PRM-LCM group; N.S., *p* > 0.05 Li-Pilo-LCM group vs. Li-Pilo-PRM-LCM group.

In the Y-maze task, the Li-Pilo-veh group showed a significant decrease in the percentage of spontaneous alternations (SA) compared to the C-veh animals (31.17 ± 7.13 vs. 65.10 ± 4.66, *p* = 0.001) (Figure 3). Both groups, Li-Pilo-PRM and Li-Pilo-LCM, significantly increased spontaneous alternations compared to the animals with a model of epilepsy (69.29 ± 4.67 vs. 31.17 ± 7.13, *p* < 0.001 and 67.89 ± 6.63 vs. 31.17 ± 7.13, *p* < 0.001, respectively). The same positive effect on SA was observed in the group treated with both drugs (Li-Pilo-PRM-LCM) compared to the Li-Pilo-veh animals (58.34 ± 3.27 vs. 31.17 ± 7.13, *p* = 0.012). No statistical significance was observed when comparing the C-veh group and the three groups treated singly with PRM and LCM or in a combination of both drugs, *p* > 0.05. In addition, no difference was detected between the Li-Pilo-PRM and Li-Pilo-LCM groups when compared to the animals treated with both drugs (Li-Pilo-PRM-LCM), *p* > 0.05.

### 3.3. One-Way Step-Through Passive Avoidance Test with Negative Reinforcement

**Figure 4:**
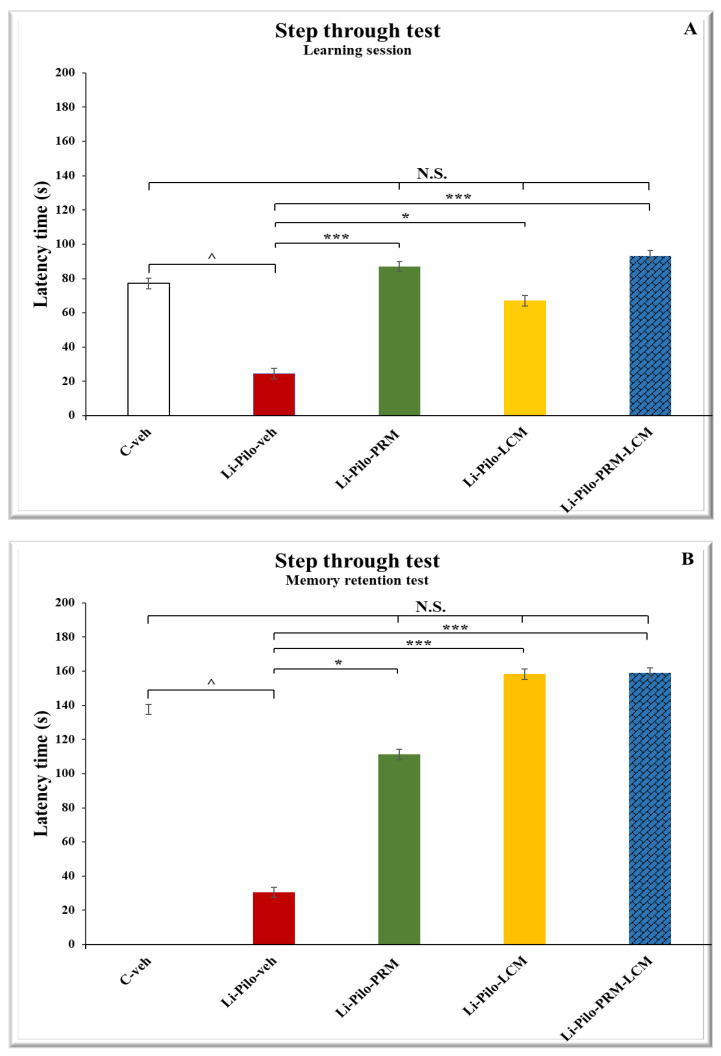
(**A**). Effect of long-term treatment with perampanel (PRM) and lacosamide (LCM), singly or in combination, on the latency time (s) during a learning session in the step-through passive avoidance test in animals with a model of temporal lobe epilepsy. ^^^ *p* < 0.05 Li-Pilo-veh vs. C-veh group; *** *p* < 0.001 Li-Pilo-PRM vs. Li-Pilo-veh group; * *p* < 0.05 Li-Pilo-LCM vs. Li-Pilo-veh group; *** *p* < 0.001 Li-Pilo-PRM vs. Li-Pilo-veh group; no significance (N.S.), *p* > 0.05 C-veh group vs. Li-Pilo-PRM group; *p* > 0.05 C-veh group vs. Li-Pilo-LCM group; *p* > 0.05 C-veh group vs. Li-Pilo-PRM-LCM group; N.S., *p* > 0.05 Li-Pilo-PRM vs. Li-Pilo-PRM-LCM group; N.S., *p* > 0.05 Li-Pilo-LCM group vs. Li-Pilo-PRM-LCM group. (**B**). Effect of long-term treatment with perampanel (PRM) and lacosamide (LCM), singly or in combination, on the latency time (s) during a memory retention test in the step-through passive avoidance test in animals with a model of temporal lobe epilepsy. ^^^ *p* < 0.05 Li-Pilo-veh vs. C-veh group; * *p* < 0.001 Li-Pilo-PRM vs. Li-Pilo-veh group; *** *p* < 0.001 Li-Pilo-LCM vs. Li-Pilo-veh group; *** *p* < 0.001 Li-Pilo-PRM-LCM vs. Li-Pilo-veh group; no significance (N.S.), *p* > 0.05 C-veh group vs. Li-Pilo-PRM group; *p* > 0.05 C-veh group vs. Li-Pilo-LCM group; *p* > 0.05 C-veh group vs. Li-Pilo-PRM-LCM group; N.S., *p* > 0.05 Li-Pilo-PRM vs. Li-Pilo-PRM-LCM group; N.S., *p* > 0.05 Li-Pilo-LCM group vs. Li-Pilo-PRM-LCM group.

The post hoc test revealed that the Li-Pilo animals showed a decreased reaction time during the learning session (24.51 ± 5.83 vs. 77.13 ± 4.84, *p* = 0.007) and the memory retention test compared to the C-veh group (30.60 ± 6.61 vs. 137.58 ± 21.80, *p* = 0.001) (Figure 4A,B). The comparison between the Li-Pilo-veh rats and the group with a model of epilepsy treated with PRM showed that the animals treated with the anticonvulsant significantly increased their step-through latency time during both sessions on day 1 (86.98 ± 10.99 vs. 24.51 ± 5.83, *p* = 0.001) and day 2 (111.21 ± 25.41 vs. 30.60 ± 6.61, *p* = 0.023). The Li-Pilo-LCM group also showed an increase in the time for entering the dark compartment during the learning session (67.01 ± 15.49 vs. 24.51 ± 5.83, *p* = 0.041) and the memory retention test compared to the animals with a model of TLE (158.20 ± 19.80 vs. 30.60 ± 6.61, *p* < 0.001). Moreover, during the two experimental days, the group that was treated with both drugs showed a significant increase in the latency time compared to the Li-Pilo-veh animals (93.27 ± 9.95 vs. 24.51 ± 5.83, *p* < 0.001 for day 1 and 158.25 ± 6.28 vs. 30.60 ± 6.61, *p* < 0.001 for day 2). No statistical significance was observed when comparing the C-veh group and the three groups treated singly with PRM and LCM or a combination of both drugs, *p* > 0.05. Moreover, no difference was detected as well between the single-drug-treated groups when compared to the animals treated with both drugs (Li-Pilo-PRM-LCM), *p* > 0.05 during the learning session and memory retention test.

### 3.4. Elevated Plus Maze (EPM) Test

**Figure 5:**
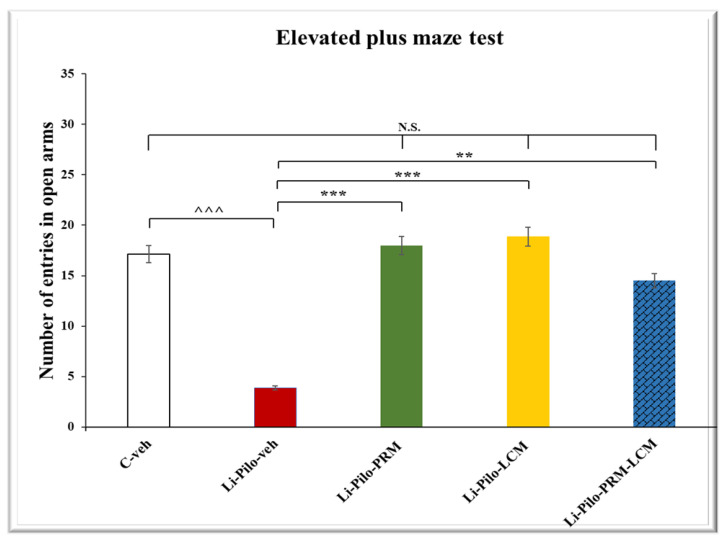
Effect of long-term treatment with perampanel (PRM) and lacosamide (LCM), singly or in combination, on the number of entries in open arms in the elevated plus maze test in animals with a model of temporal lobe epilepsy. ^^^^^ *p* < 0.001 Li-Pilo-veh vs. C-veh group; *** *p* < 0.001 Li-Pilo-PRM vs. Li-Pilo-veh group; *** *p* < 0.001 Li-Pilo-LCM vs. Li-Pilo-veh group; ** *p* < 0.01 Li-Pilo-PRM-LCM vs. Li-Pilo-veh group; no significance (N.S.), *p* > 0.05 C-veh group vs. Li-Pilo-PRM group; *p* > 0.05 C-veh group vs. Li-Pilo-LCM group; *p* > 0.05 C-veh group vs. Li-Pilo-PRM-LCM group; *p* > 0.05 Li-Pilo-PRM vs. Li-Pilo-PRM-LCM group; *p* > 0.05 Li-Pilo-LCM group vs. Li-Pilo-PRM-LCM group.

**Figure 6:**
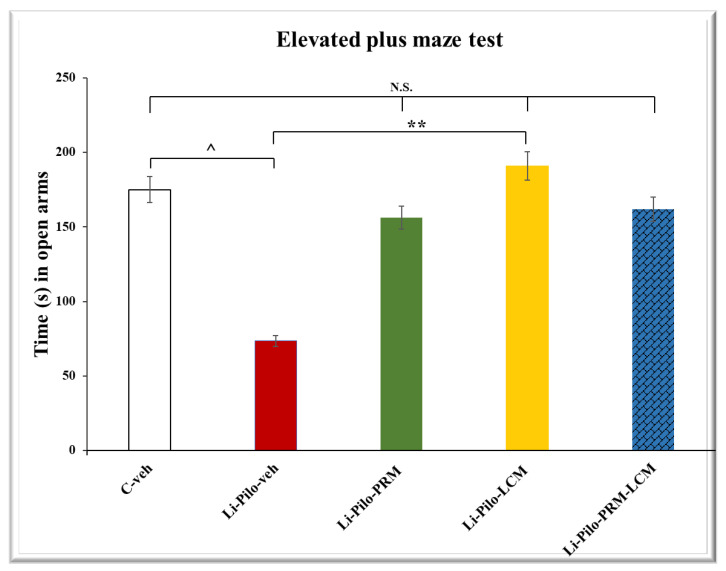
Effect of long-term treatment with perampanel (PRM) and lacosamide (LCM), singly or in combination, on the time (s) spent in open arms in the elevated plus maze test (300 s total time) in animals with a model of temporal lobe epilepsy. ^^^ *p* < 0.05 Li-Pilo-veh vs. C-veh group; ** *p* < 0.01 Li-Pilo-LCM vs. Li-Pilo-veh group; no significance (N.S.), *p* > 0.05 C-veh group vs. Li-Pilo-PRM group; *p* > 0.05 C-veh group vs. Li-Pilo-LCM group; *p* > 0.05 C-veh group vs. Li-Pilo-PRM-LCM group; *p* > 0.05 Li-Pilo-PRM vs. Li-Pilo-PRM-LCM group; *p* > 0.05 Li-Pilo-LCM group vs. Li-Pilo-PRM-LCM group.

**Figure 7:**
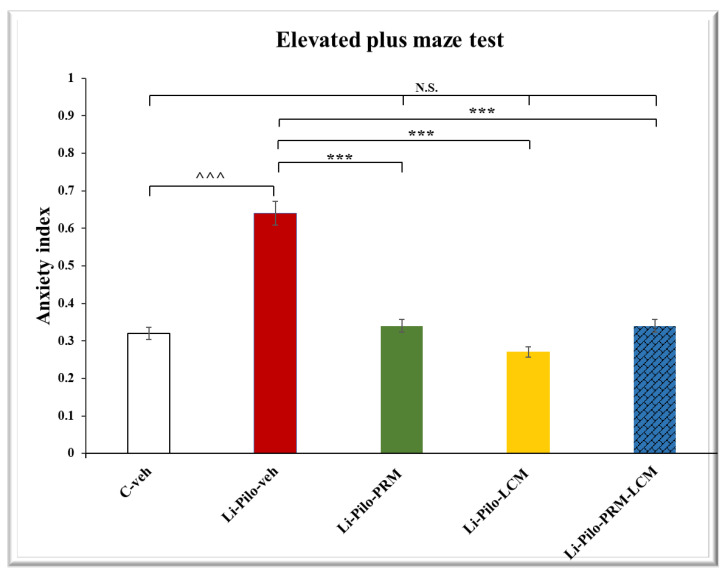
Effect of long-term treatment with perampanel (PRM) and lacosamide (LCM), singly or in combination, on the anxiety index in the elevated plus maze test in animals with a model of temporal lobe epilepsy. ^^^^^ *p* < 0.001 Li-Pilo-veh vs. C-veh group; *** *p* < 0.001 Li-Pilo-PRM vs. Li-Pilo-veh group; *** *p* < 0.001 Li-Pilo-LCM vs. Li-Pilo-veh group; *** *p* < 0.001 Li-Pilo-PRM-LCM vs. Li-Pilo-veh group; no significance (N.S.), *p* > 0.05 C-veh group vs. Li-Pilo-PRM group; *p* > 0.05 C-veh group vs. Li-Pilo-LCM group; *p* > 0.05 C-veh group vs. Li-Pilo-PRM-LCM group; *p* > 0.05 Li-Pilo-PRM vs. Li-Pilo-PRM-LCM group; *p* > 0.05 Li-Pilo-LCM group vs. Li-Pilo-PRM-LCM group.

In the EPM test, both groups, Li-Pilo-PRM and Li-Pilo-LCM, had a higher number of entries in the open arms compared to the animals with a model of TLE (*p* < 0.001, respectively) (Figure 5). The group treated with both drugs, PRM and LCM, also showed an increased number of entries in comparison with the Li-Pilo animals (*p* = 0.006). The post hoc test demonstrated that only the Li-Pilo-LCM group had a significantly longer time in the aversive area compared to the Li-Pilo-veh group (*p* = 0.009) (Figure 6). The Li-Pilo-veh group showed a higher anxiety index compared to the C-veh animals (*p* < 0.001). The three groups treated with the drugs singly (Li-Pilo-PRM and Li-Pilo-LCM) or in combination of both (Li-Pilo-PRM-LCM) had lower anxiety index compared to the animals with a model of TLE (*p* < 0.001, *p* < 0.001, and *p* = 0.001, respectively) (Figure 7). The mean values and SEM found in the EPM test are presented in Supplementary Table S1.

### 3.5. BDNF Immunohistochemical Expression

**Figure 8:**
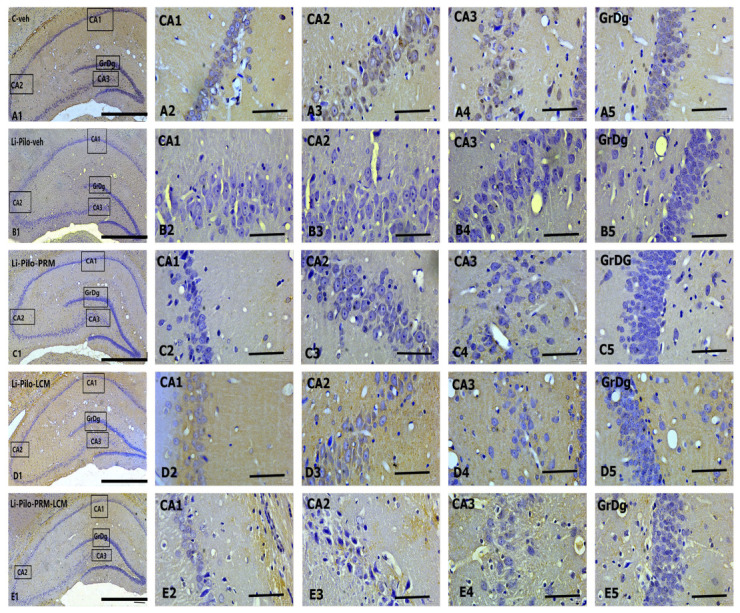
Immunohistochemical expression of BDNF in the dorsal hippocampus. C-veh group (**A1**–**A5**), Li-Pilo-veh group (**B1**–**B5**), Li-Pilo-PRM group (**C1**–**C5**), Li-Pilo-LCM group (**D1**–**D5**), Li-Pilo-PRM-LCM group (**E1**–**E5**). Higher magnifications of the rectangles in all five groups are given. Scale bars = 200 µm (**A1**,**B1**,**C1**,**D1**,**E1**); 50 µm (**A2**–**A5**,**B2**–**B5**,**C2**–**C5**,**D2**–**D5**,**E2**–**E5**).

Immunohistochemical localization of BDNF in the dorsal hippocampus (Figure 8A–E) in cornu ammonis (CA1, CA2, and CA3) subfields, as well as in the granular cell layer in the dentate gyrus (GrDG).

Representative images showing BDNF immunostaining in the control C-veh group (A1–A5), Li-Pilo-veh group (B1–B5), Li-Pilo-PRM group (C1–C5), Li-Pilo-LCM group (D1–D5), and Li-Pilo-PRM-LCM group (E1–E5). Higher magnifications of the rectangles in all five groups are given. The immune response to BDNF in the C-veh group is moderate but strongly reduced in the Li-Pilo-veh group, especially in CA3 hippocampal fields (A4, B4). In the groups of experimental rats treated with antiepileptic drugs (Li-Pilo-PRM, Li-Pilo-LCM, and Li-Pilo-PRM-LCM group), we observed a significant improvement in BDNF levels, again, most detectable in the CA3 subfield (C4, D4, and E4) compared to the control group of rats undergoing epileptic seizures.

**Figure 9:**
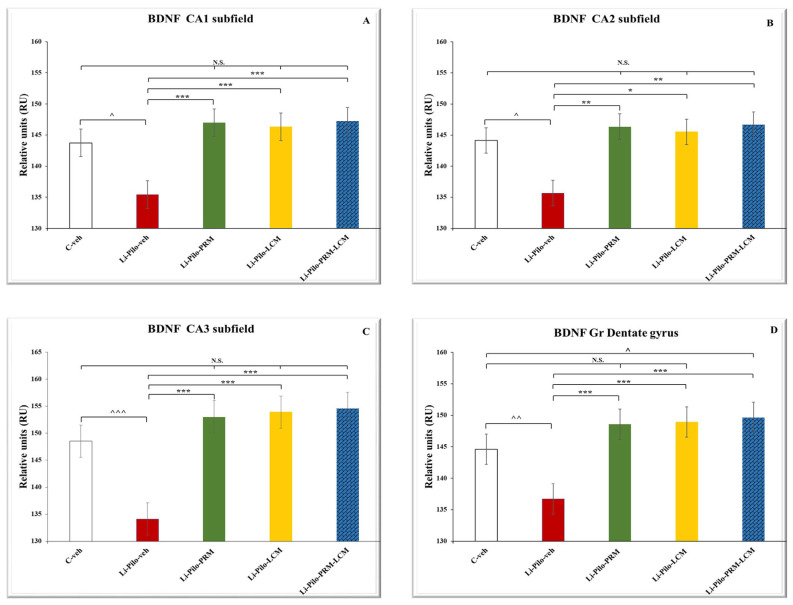
Effect of long-term treatment with perampanel (PRM) and lacosamide (LCM), singly or in combination, on the BDNF levels (RU) in animals with a model of temporal lobe epilepsy: (**A**) in the hippocampal CA1 subfield, ^^^ *p* < 0.05, Li-Pilo-veh vs. C-veh group; *** *p* < 0.001 Li-Pilo-PRM vs. Li-Pilo-veh group; *** *p* < 0.001 Li-Pilo-LCM vs. Li-Pilo-veh group; *** *p* < 0.001 Li-Pilo-PRM-LCM vs. Li-Pilo-veh group; no significance (N.S.), *p* > 0.05 C-veh group vs. Li-Pilo-PRM group; *p* > 0.05 C-veh group vs. Li-Pilo-LCM group; *p* > 0.05 C-veh group vs. Li-Pilo-PRM-LCM group; *p* > 0.05 Li-Pilo-PRM vs. Li-Pilo-PRM-LCM group; *p* > 0.05 Li-Pilo-LCM group vs. Li-Pilo-PRM-LCM group. (**B**) in the hippocampal CA2 subfield, ^^^ *p* < 0.05, Li-Pilo-veh vs. C-veh group; ** *p* < 0.01 Li-Pilo-PRM vs. Li-Pilo-veh group; * *p* < 0.05 Li-Pilo-LCM vs. Li-Pilo-veh group; ** *p* < 0.01 Li-Pilo-PRM-LCM vs. Li-Pilo-veh group; no significance (N.S.), *p* > 0.05 C-veh group vs. Li-Pilo-PRM group; *p* > 0.05 C-veh group vs. Li-Pilo-LCM group; *p* > 0.05 C-veh group vs. Li-Pilo-PRM-LCM group; *p* > 0.05 Li-Pilo-PRM vs. Li-Pilo-PRM-LCM group; *p* > 0.05 Li-Pilo-LCM group vs. Li-Pilo-PRM-LCM group; (**C**) in the hippocampal CA3 subfield, ^^^^^ *p* < 0.001, Li-Pilo-veh vs. C-veh group; *** *p* < 0.001 Li-Pilo-PRM vs. Li-Pilo-veh group; *** *p* < 0.001 Li-Pilo-LCM vs. Li-Pilo-veh group; *** *p* < 0.001 Li-Pilo-PRM-LCM vs. Li-Pilo-veh group; no significance (N.S.), *p* > 0.05 C-veh group vs. Li-Pilo-PRM group; *p* > 0.05 C-veh group vs. Li-Pilo-LCM group; *p* > 0.05 C-veh group vs. Li-Pilo-PRM-LCM group; *p* > 0.05 Li-Pilo-PRM vs. Li-Pilo-PRM-LCM group; *p* > 0.05 Li-Pilo-LCM group vs. Li-Pilo-PRM-LCM group and (**D**) in the granular cell layer in the dentate gyrus, ^^^^ *p* < 0.01, Li-Pilo-veh vs. C-veh group; *** *p* < 0.001 Li-Pilo-PRM vs. Li-Pilo-veh group; *** *p* < 0.001 Li-Pilo-LCM vs. Li-Pilo-veh group; *** *p* < 0.001 Li-Pilo-PRM-LCM vs. Li-Pilo-veh group; ^^^ *p* < 0.05 Li-Pilo-PRM-LCM vs. C-veh group; no significance (N.S.), *p* > 0.05 C-veh group vs. Li-Pilo-PRM group; *p* > 0.05 C-veh group vs. Li-Pilo-LCM group; *p* > 0.05 Li-Pilo-PRM vs. Li-Pilo-PRM-LCM group; *p* > 0.05 Li-Pilo-LCM group vs. Li-Pilo-PRM-LCM group.

The post hoc test demonstrated that only the Li-Pilo animals showed a significant decrease in the BDNF expression in the CA1 (cornu ammonis 1) subfield of the hippocampus in comparison with the C-veh group (*p* = 0.002) (Figure 9A). The three experimental groups with a model of TLE, which are treated with PRM/LCM or both drugs, significantly increased the expression of the neurotrophic factor in the CA1 region compared to the Li-Pilo-veh animals (*p* < 0.001, respectively).

In the CA2 subfield of the hippocampus, the Li-Pilo-veh group showed a significant decrease in the BDNF expression compared to the saline animals (*p* = 0.04) (Figure 9B). Chronic treatment with both groups, Li-Pilo-PRM and the one with the drug combination Li-Pilo-PRM-LCM, showed a significant increase in the neurotropic expression compared to the animals with a model of TLE (*p* = 0.006 and *p* = 0.004). The Li-Pilo-LCM group also showed higher expression of BDNF in the CA2 subfield in comparison with the animals with a model of TLE (*p* = 0.012).

The post hoc test revealed that the animals with epilepsy had a significantly lower BDNF expression in comparison with the C-veh group in the CA3 region of the hippocampus (*p* < 0.001) (Figure 9C). The three experimental groups treated with both drugs, PRM and LCM, singly or in combination, showed an increase in the expression of the neurotrophic factor compared to the Li-Pilo-veh rats in the same region (*p* < 0.001, respectively).

No statistical significance of the neurotrophic factor expression in the three subfields CA1, CA2, and CA3 was observed when comparing the C-veh group and the three groups treated singly with PRM and LCM or in combination with both drugs, *p* > 0.05. In addition, no difference was detected between the singly treated groups with PRM and LCM when compared to the animals treated with both drugs (Li-Pilo-PRM-LCM), *p* > 0.05.

The analyses of variance revealed that the Li-Pilo-veh group showed a significant decrease in the BDNF expression in the DG compared to the vehicle animals (*p* < 0.01), while only the Li-Pilo-PRM-LCM rats showed an increase in the expression of the neurotrophic factor compared to the C-veh group (*p* = 0.022) (Figure 9D). A tendency for a similar effect was also observed by the Li-Pilo-LCM animals (*p* = 0.068). All three groups treated with the ASMs—PRM and LCM singly or in combination with both—managed to increase the BDNF expression in the DG in comparison with the Li-Pilo animals (*p* < 0.001, respectively). No statistical significance of the neurotrophic factor expression in the DG was observed when comparing the C-veh group and the animals treated singly with PRM and LCM, *p* > 0.05. The mean values and S.E.M., found for BDNF expression, are presented in Supplementary Table S2.

### 3.6. Cyclin D1 Immunohistochemical Expression

**Figure 10:**
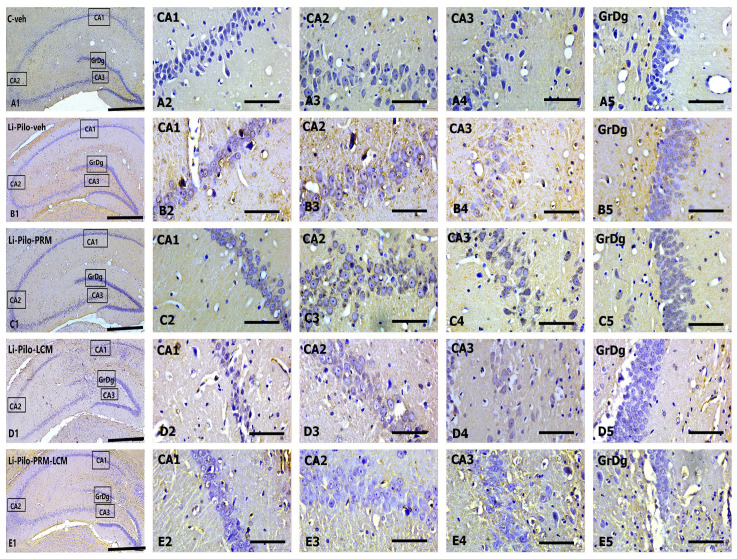
(**A**–**E**). Immunohistochemical expression of Cyclin D1 in the dorsal hippocampus. C-veh group (**A1**–**A5**), Li-Pilo-veh group (**B1**–**B5**), Li-Pilo-PRM group (**C1**–**C5**), Li-Pilo-LCM group (**D1**–**D5**), Li-Pilo-PRM-LCM group (**E1**–**E5**). Higher magnifications of the rectangles in all five groups are given. Scale bars = 200 µm (**A1**,**B1**,**C1**,**D1**,**E1**); 50 µm (**A2**–**A5**,**B2**–**B5**,**C2**–**C5**,**D2**–**D5**,**E2**–**E5**).

Immunohistochemical localization of Cyclin D1 in the dorsal hippocampus (Figure 10A–E) in the cornu ammonis (CA1, CA2, and CA3) subfields, including in the dentate gyrus, and granular cell layer (GrDg).

Representative images showing Cyclin D1 immunoexpression in the control C-veh group (A1–A5), Li-Pilo-veh group (B1–B5), Li-Pilo-PRM group (C1–C5), Li-Pilo-LCM group (D1–D5), and Li-Pilo-PRM-LCM group (E1–E5). The insets show a higher magnification in the granular layer of the dentate gyrus and the three parts of the cornu ammonis hippocampal region. The immune response to Cyclin D1 is the most significant in the Li-Pilo-veh group, especially in the CA3 hippocampal fields (B4). In all experimental groups treated with antiepileptic drugs (Li-Pilo-PRM, Li-Pilo-LCM, and Li-Pilo-PRM-LCM groups), a significant decrease in Cyclin D1 levels was shown, again most detectable in the CA3 subfield (C4, D4, and E4).

**Figure 11:**
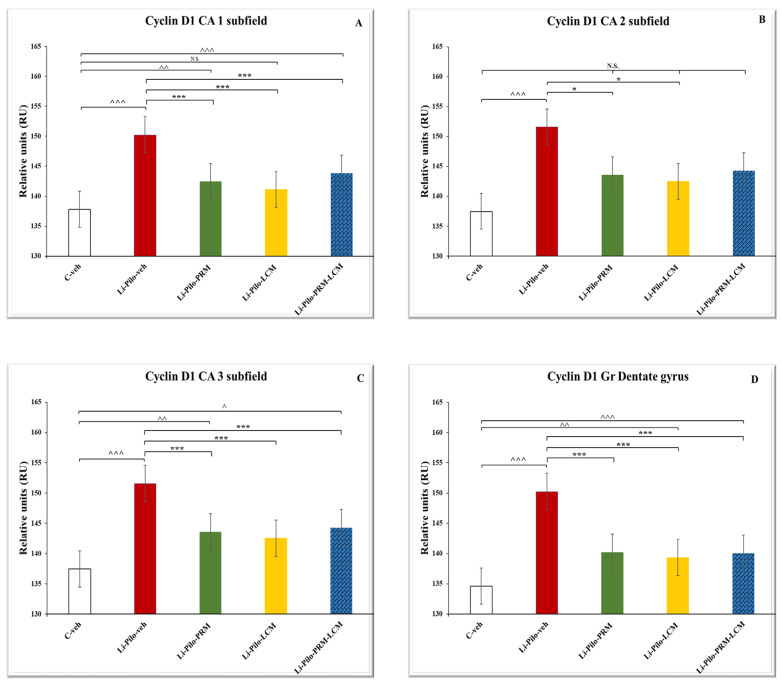
Effect of long-term treatment with perampanel (PRM) and lacosamide (LCM), singly or in combination, on the Cyclin D1 levels (RU) in (**A**) the hippocampal CA1 subfield, ^^^^^ *p* < 0.001 Li-Pilo-veh vs. C-veh group, ^^^^ *p* < 0.01 Li-Pilo-PRM vs. C-veh group, *** *p* < 0.001 Li-Pilo-PRM vs. Li-Pilo-veh group; *** *p* < 0.001 Li-Pilo-LCM vs. Li-Pilo-veh group; ^^^^^ *p* < 0.001 Li-Pilo-PRM-LCM vs. C-veh group, *** *p* < 0.001 Li-Pilo-PRM-LCM vs. Li-Pilo-veh group; no significance (N.S.), *p* > 0.05 C-veh group vs. Li-Pilo-LCM group, *p* > 0.05 Li-Pilo-PRM vs. Li-Pilo-PRM-LCM group; *p* > 0.05 Li-Pilo-LCM group vs. Li-Pilo-PRM-LCM group (**B**) CA2 subfield, ^^^^^ *p* < 0.001 Li-Pilo-veh vs. C-veh group, * *p* < 0.05 Li-Pilo-PRM vs. Li-Pilo-veh group; * *p* < 0.05 Li-Pilo-LCM vs. Li-Pilo-veh group; N.S., *p* > 0.05 C-veh group vs. Li-Pilo-PRM group; *p* > 0.05 C-veh group vs. Li-Pilo-LCM group; *p* > 0.05 C-veh group vs. Li-Pilo-PRM-LCM group; *p* > 0.05 Li-Pilo-PRM vs. Li-Pilo-PRM-LCM group; *p* > 0.05 Li-Pilo-LCM group vs. Li-Pilo-PRM-LCM group; *p* > 0.05 Li-Pilo-PRM-LCM vs. Li-Pilo-veh (**C**) CA3 subfield, ^^^^^ *p* < 0.001 Li-Pilo-veh vs. C-veh group; ^^^^^ *p* < 0.01 Li-Pilo-PRM vs. C-veh group; ^^^ *p* < 0.05, ^^^^ *p* < 0.01 Li-Pilo-LCM vs. C-veh group; ^^^ *p* < 0.05, ^^^^^ *p* < 0.001 Li-Pilo-PRM-LCM vs. C-veh group, *** *p* < 0.001 Li-Pilo-PRM vs. Li-Pilo-veh group; ^^^ *p* < 0.05, Li-Pilo-LCM vs. C-veh group; *** *p* < 0.001 Li-Pilo-LCM vs. Li-Pilo-veh group; ^^^ *p* < 0.05 Li-Pilo-PRM-LCM vs. C-veh group; *** *p* < 0.001 Li-Pilo-PRM-LCM vs. Li-Pilo-veh group and (**D**) in the granular cell layer in the dentate gyrus, ^^^^^ *p* < 0.001 Li-Pilo-veh vs. C-veh group; ^^^^^ *p* < 0.001 Li-Pilo-PRM vs. C-veh group; *** *p* < 0.001 Li-Pilo-PRM vs. Li-Pilo-veh group; ^^^^ *p* < 0.01 Li-Pilo-LCM vs. C-veh group; *** *p* < 0.001 Li-Pilo-LCM vs. Li-Pilo-veh group; ^^^^^ *p* < 0.001 Li-Pilo-PRM-LCM vs. C-veh group; *** *p* < 0.001 Li-Pilo-PRM-LCM vs. Li-Pilo-veh group.

The statistical analyses in the CA1 subfield of the hippocampus revealed that the animals with a model of TLE showed a significant increase in the Cyclin D1 expression (*p* < 0.001). The same effect was observed in the Li-Pilo-PRM and Li-Pilo-PRM-LCM animals when compared with the C-veh group (*p* = 0.005, *p* < 0.001, respectively) (Figure 11A). In contrast, all three groups with a model of epilepsy and treated with PRM/LCM singly or in combination showed a decrease in the cyclin expression compared to the Li-Pilo-veh animals (*p* < 0.001, respectively).

The post hoc test revealed that the animals with a model of epilepsy showed a significant overexpression of Cyclin D1 in the CA2 hippocampal region compared to the C-veh rats (*p* < 0.001) (Figure 11B). A decrease in cyclin expression was demonstrated by both groups with a model of TLE and treated with PRM and LCM in comparison with the Li-Pilo-veh animals (*p* = 0.033 and *p* = 0.012, respectively). A tendency for such an effect was demonstrated by the group treated with both drugs (*p* = 0.061).

In the CA 3 hippocampal subfield, the Li-Pilo-veh animals had significantly higher Cyclin D1 protein expression (*p* < 0.001), while the opposite effect was observed in the three groups, Li-Pilo-PRM (*p* = 0.002), Li-Pilo-LCM (*p* = 0.017), and Li-Pilo-PRM-LCM (*p* = 0.031), compared to the C-veh rats (Figure 11C). Moreover, the same three experimental groups with a model of epilepsy and treated with drugs showed a decrease in the expression of the apoptotic factor compared to the Li-Pilo-veh animals (*p* < 0.001).

The analyses of variance demonstrated that the Li-Pilo-veh group showed a significant increase in the Cyclin D1 expression in the GrDG compared to the vehicle animals (*p* < 0.001). The same effect was observed in the three groups treated with the drugs singly or in combination (*p* < 0.001, Li-Pilo-PRM group compared to the C-veh group; *p* = 0.002, Li-Pilo-LCM group compared to the C-veh group, and *p* < 0.001, Li-Pilo-PRM-LCM group compared to the C-veh group) (Figure 11D). In contrast, the same three experimental groups, Li-Pilo-PRM, Li-Pilo-LCM, and Li-Pilo-PRM-LCM, showed significantly lower Cyclin D1 expression in this region in comparison with the Li-Pilo animals (*p* < 0.001, respectively).

In addition, no significant difference was detected between the singly treated groups with PRM and LCM when compared to the animals treated with both drugs (Li-Pilo-PRM-LCM), *p* > 0.05 on the Cyclin D1 hippocampal expression in all subfields and in the granular cell layer of the DG (Figure 11). The mean values and S.E.M., found for Cyclin D1 expression, are presented in Supplementary Table S3.

## 4. Discussion

The current study was conducted to investigate the tolerability and effectiveness of a single long-term administration and the combination of two third-generation ASMs with unique mechanisms of action of PRM and LCM. We found that the rats with the model of post-SE-induced TLE exerted various comorbidities such as impairment of different cognitive domains and exploratory behavior. In addition, the two anticonvulsants, PRM and LCM, administered singly in high doses, 3 mg/kg and 30 mg/kg, respectively, during the epileptogenesis and chronic state of the model of TLE, reversed the behavioral changes induced by SE. The combination of both drugs, administered in low doses of PRM (0.5 mg/kg) and LCM (3 mg/kg), demonstrated similar anxiolytic and cognitive-improving effects compared to the singly administered drugs in high doses. Moreover, in the animals with a model of epilepsy, an activated process of apoptosis accompanied by reduced levels of the neurotrophic factor was detected. In contrast, we observed a significant improvement in the neurotrophic factor BDNF levels and decreased expression of the apoptotic factor Cyclin D1 by the three groups treated with ASMs singly or in combination. All these data suggest a disease-modifying effect of both drugs, which could be closely related to their cognitive-enhancing effect.

Long-term neurophysiological deficits associated with epilepsy are strongly influenced by extrinsic factors, such as early-onset epilepsy, SE high frequency, poor seizure control, multiple polytherapy, etc. [40]. All these factors lead to progressive changes in brain connectivity, resulting in cognitive decline [40,41,42]. In line with other findings, in our study, the animals with a model of TLE showed impaired spatial working memory, deteriorated passive learning and memory, and decreased exploratory behavior. In contrast, long-term treatment with PRM singly at a dose of 3 mg/kg managed to weaken the negative effect of epilepsy and improve passive learning, facilitating the formation of spatial working memory and long-term memory traces. PRM enhanced exploratory behavior, although the drug failed to improve object recognition retrieval. Our data are in agreement with other experimental studies that have found that PRM preserves spatial and recognition memory as a result of the slowed-down appearance of spontaneous motor seizure activity and the reduced SE-induced hippocampal cell death [43]. In compliance with our results is the experimental model of ischemia where PRM (1.5 mg/kg) promotes memory consolidation and retrieval as a result of anti-inflammatory, antioxidant, and anti-apoptotic activities [44]. In the study of Martins et al. [45], the drug promotes spatial cognitive performance, and the observed result is due to the stimulation of GAP-43 expression, an essential protein for the neurotrophic effects of BDNF. Moreover, Aida et al. [46] demonstrated in an experimental model of traumatic brain injury that pre- and post-PRM treatment attenuates the increased hippocampal pro-apoptotic bax/bcl-xL ratio and reduces learning and memory deficits assessed by the Morris water maze test in male adult rats. Multiple lines of evidence suggest that different brain regions are associated with spatial and passive memory, namely the hippocampus, amygdala, and prefrontal cortex [47].

In the present study, we found that long-term LCM treatment (30 mg/kg) improved both spatial consolidation and retrieval and facilitated passive learning and formation of memory traces in an experimental lithium–pilocarpine model of TLE. These results are in agreement with our previous data in a similar model [34] and with other researchers who have found neuroprotective and cognitive-improving effects of the drug in different experimental models of hypoxic–ischemic brain injury [48], lipopolysaccharide-induced neuroinflammation [49], and traumatic spinal cord injury [50]. The authors associate most of these beneficial effects of LCM with antioxidant and anti-inflammatory activities, attenuated glial cell activation, and inhibited apoptosis in different brain regions, including the hippocampus, cortex, and cerebellum.

To the best of our knowledge, no study has investigated the long-term effect of the combination of low doses of PRM (0.5 mg/kg) and LCM (3 mg/kg) on cognitive performance and the underlying mechanisms of the observed effects during the chronic phase of an experimental model of TLE. We found that both drugs administered in low doses produced similar effects compared to the singly administered drugs in high doses, PRM (3 mg/kg) and LCM (30 mg/kg). Drug combination improved spatial working memory as well as passive learning and the formation of long-term memory. The two ASMs administered together managed to improve recognition memory while PRM failed to facilitate its consolidation and retrieval. These results complement other studies that have found a synergistic effect of PRM and zonisamide combination in a model of rat amygdala kindling of TLE [13]. The authors found a pronounced threshold increase when zonisamide administration is combined with different PRM doses. Moreover, better performance on the Rotarod test for assessing motor function was revealed for the drug combination. Additionally, it is important to note that zonisamide is an ASM and has a similar mechanism of action as LCM through influencing the slow inactivation of voltage-gated sodium channels [51]. The cognitive-enhancing effects of both drugs and their combination could be a real advantage when compared to many other anticonvulsants, especially the older ones, which usually lead to additional cognitive impairment in epileptic patients.

Anxiety is among the most common psychiatric comorbidity conditions that occur in patients with epilepsy [52]. Increased stress or anxiety can be a result of the disease process but, on the other hand, can also cause disease exacerbation. In the present study, we found that animals with a model of TLE had less time spent in the aversive area of the maze, and increased anxiety-like behavior was detected compared to the control rats. In contrast, both drugs administered singly or in combination led to a pronounced anxiolytic effect. Although data about the effect of PRM on levels of anxiety is quite scarce, a few recently published studies are in agreement with our results. The authors have demonstrated the anxiolytic activity of PRM in naïve rats [53], in mice subjected to maximal electroshock seizures [54], and in pentylenetetrazole-kindled mice [55]. The anxiolytic effect of LCM in the current study is in agreement with single-source data showing decreased levels of anxiety in patients with epilepsy [18,56,57,58].

Numerous molecular mechanisms could be underlying the pathogenesis of epilepsy and can be influenced by the administered drug therapy. As BDNF is an important neuronal modulator, regulating many critical aspects in the ontogenesis of neurons and the development of synapses [14], we investigated its hippocampal expression. Our results demonstrated impaired neuronal expression of the neurotrophic factor in the control group of animals undergoing seizures. These results are in accordance with other studies, showing that reduced hippocampal BDNF expression has been linked to impaired synaptic connections, inhibited process of neurogenesis, and cognitive impairment [16,59,60,61,62]. Identical manifestations of this neurotrophin are observed in activated microglia as well as in astrocytes. These results are in line with previous research, which considered BDNF as the most important signaling molecule between neurons and glia, especially in conditions of neuronal damage and cellular stress [19]. In our experiment, the two ASMs, PRM and LCM, administered singly or in a low-dose combination, led to a significant improvement of BDNF levels in all subfields of the hippocampus as well as in the GrDG. There is scarce data about the effect of different ASMs on the BDNF levels. Our results are in line with other studies revealing that combined drug interactions may affect, at least partially, the normalization of BDNF levels in the stressed hippocampus [61]. A recent clinical study revealed that serum BDNF levels are significantly increased in patients with epilepsy who have received PRM or valproate treatment [63]. In contrast, experimental studies reveal that some traditional ASMs like phenobarbital, topiramate, and lamotrigine can reduce mRNA levels of BDNF and lead to cognitive deficits [64]. The expression of the neurotrophic factor is increased during all processes of learning and memory in the hippocampus, which is related to improved cognitive function [16,65,66]. The neuroprotective microglia-neuron physical interactions lead to the preservation of homeostasis in the CNS and, especially, in the hippocampus [67].

Recent evidence reveals that epileptic seizures and the intake of certain medications cause neuronal damage, leading to apoptotic changes in hippocampal cells and arrest of the cell cycle. Therefore, the study of the expression of the apoptotic protein Cyclin D1 would determine the severity and direction of programmed cell death and could detect probable neuronal damage with the presence of apoptotic cell changes [27,28]. In addition, the correlation between neurotrophic and pro-apoptotic factors in the hippocampus has been established for its ontogenesis but has not yet been sufficiently studied. Moreover, there is a lack of information about the effect of different ASMs on Cyclin D1 expression.

According to Timsit et al. [27], Cyclin D1 is expressed histochemically in damaged neurons of the hippocampus. Our results on the activity of cyclin D1, which has the highest expression in the control group undergoing only epileptic seizures, are in line with the above-mentioned study. On the other hand, we observed a significant reduction in protein expression in all hippocampal subfields (CA1, CA2, and CA3, as well as in GrDG) in the three groups treated with both ASMs, PRM and LCM, singly or in combination. These results confirm the hypothesis that neuronal damage triggers the release of cell cycle proteins, resulting in increased cell death [22,24,25]. Furthermore, neurodegenerative manifestations are accompanied by microglial dystrophy, which, in turn, enhances neuronal dystrophy [68,69]. Significant expression of Cyclin D1 in astrocytic glia has also been reported [28,70], with neuron-astrocyte metabolic coupling given a crucial role in learning and long-term memory [28,71]. All mentioned studies determine the Cyclin D1 manifestation exclusively in cells that undergo apoptotic changes after ischemic damage or epileptic seizures. Therefore, many authors define Cyclin D1 as a kind of marker for the occurrence of programmed cell death [27,72,73]. The consequences of epileptic damage in the hippocampus are most significant in the vulnerable CA3 region, which is in line with our results [27,74,75]. The correlation between neurotrophic and apoptotic factors in the hippocampus is also different at different stages of injury. Cell signaling in the epileptic hippocampus is defective at the beginning of the damage, while the neurotrophic factors do not have a significant effect in the initial phases of the injury, but they are leading at a later stage, especially in the granular cells of the dentate gyrus [37]. This could be an important mechanism through which ASMs, which increase BDNF levels, improve cognitive functions in epileptic patients.

A limitation of the present study is the use of only male rats. The conduction of the experiment with female rats could be a subject of future research, as memory, anxiety levels, as well as the BDNF and Cyclin D1 expression, can be influenced by the natural fluctuations in the sex hormone levels during the ovarian cycle in female rats.

## 5. Conclusions

In conclusion, the present study demonstrates that chronic treatment with perampanel and lacosamide, singly in a high-dose or in a low-dose combination of both during epileptogenesis and chronic states of an experimental model of Li-Pilo TLE has pronounced cognitive-enhancing and anxiolytic effects. These beneficial outcomes could be associated with increased expression of the neurotrophic factor BDNF and decreased levels of the apoptotic factor Cyclin D1 in the neurons of the hippocampus. These additional mechanisms of both ASMs could be essential for preserving neuronal survival and homeostasis in the CNS and especially in the hippocampus, which could be an important advantage for their choice compared to many other anticonvulsants.

## Data Availability

All data reported in this paper will be shared by the lead contact upon request.

## Supplementary Materials

The following supporting information can be downloaded at: https://www.mdpi.com/article/10.3390/cimb46120838/s1, Table S1: Summary of data of EPM test on mean values ± SEM; Table S2: Summary of data of BDNF expression in the hippocampus on mean values ± SEM; Table S3: Summary of data of Cyclin D1 expression in the hippocampus on mean values ± SEM.
