# Supplementary material for: BDNF/Cyclin D1 Signaling System and Cognitive Performance After Perampanel and Lacosamide Treatment Singly or in Combination in an Experimental Model of Temporal Lobe Epilepsy
Source: Curr Issues Mol Biol. 2024 Dec 11;46(12):14010–32. doi: 10.3390/cimb46120838 (PMC11727337; doi:10.3390/cimb46120838)
Supplement: Supplementary file 1 [file cimb-46-00838-s001.zip › cimb-3323000-supplementary.pdf]

Supplement

Table S1. Summary of data of EPM test on mean values ± SEM.

Groups	Number of entries in open arms Mean ± SEM	Time (sec) in open arms Mean ± SEM	Anxiety index (AI) Mean ± SEM
C-veh	17.13 ± 1.78	175.00 ± 13.92	0.32 ± 0.038
Li-Pilo-veh	3.88 ± 1.25	73.50 ± 5.38	0.64 ± 0.028
Li-Pilo-PRM	18.00 ± 2.81	156.25 ± 7.72	0.34 ± 0.053
Li-Pilo-LCM	18.88 ± 2.42	190.75 ± 11.99	0.27 ± 0.040
Li-Pilo-PRM-LCM	14.50 ± 1.39	162.00 ± 8.79	0.34 ± 0.067

Table S2. Summary of data of BDNF expression in the hippocampus on mean values ± SEM.

Groups	BDNF CA1 Mean ± SEM	BDNF CA2 Mean ± SEM	BDNF CA3 Mean ± SEM	BDNF GrDG Mean ± SEM
C-veh	143.75 ± 1.81	144.13 ± 1.13	148.52 ± 1.99	144.59 ± 0.63
Li-Pilo-veh	135.43 ± 0.67	135.67 ± 1.06	134.12 ± 1.10	136.73 ± 0.87
Li-Pilo-PRM	146.97 ± 0.88	146.35 ± 2.23	153.03 ± 2.33	148.59 ± 0.67
Li-Pilo-LCM	146.34 ± 1.80	145.52 ± 2.57	153.92 ± 2.79	148.91 ± 1.78
Li-Pilo-PRM-LCM	147.22 ± 1.40	146.68 ± 2.44	154.49 ± 1.08	149.66 ± 1.15

Table S3. Summary of data of Cyclin D1 expression in the hippocampus on mean values ± SEM.

Groups	Cyclin D1 CA1 Mean ± SEM	Cyclin D1 CA2 Mean ± SEM	Cyclin D1 CA3 Mean ± SEM	Cyclin D1 GrDG Mean ± SEM
C-veh	137.80 ± 0.62	137.48 ± 0.43	138.83 ± 1.59	134.61 ± 0.76
Li-Pilo-veh	150.25 ± 1.27	151.59 ± 3.83	155.03 ± 0.81	150.25 ± 1.02
Li-Pilo-PRM	142.49 ± 0.79	143.60 ± 0.77	144.40 ± 0.59	140.19 ± 0.47
Li-Pilo-LCM	141.13 ± 0.72	142.52 ± 1.11	143.39 ± 0.69	139.33 ± 0.89
Li-Pilo-PRM-LCM	143.85 ± 0.73	144.29 ± 0.41	143.03 ± 0.74	140.03 ± 0.74
